# Guideline for allergological diagnosis of drug hypersensitivity reactions: S2k Guideline of the German Society for Allergology and Clinical Immunology (DGAKI) in cooperation with the German Dermatological Society (DDG), the Association of German Allergologists (ÄDA), the German Society for Pediatric Allergology (GPA), the German Contact Dermatitis Research Group (DKG), the German Society for Pneumology (DGP), the German Society of Otorhinolaryngology, Head and Neck Surgery, the Austrian Society of Allergology and Immunology (ÖGAI), the Austrian Society of Dermatology and Venereology (ÖGDV), the German Academy of Allergology and Environmental Medicine (DAAU), and the German Documentation Center for Severe Skin Reactions (dZh)

**DOI:** 10.5414/ALX02422E

**Published:** 2023-08-09

**Authors:** Knut Brockow, Gerda Wurpts, Axel Trautmann, Wolfgang Pfützner, Regina Treudler, Andreas J. Bircher, Randolph Brehler, Timo Buhl, Heinrich Dickel, Thomas Fuchs, Thilo Jakob, Julia Kurz, Burkhard Kreft, Lars Lange, Hans F. Merk, Maja Mockenhaupt, Norbert Mülleneisen, Hagen Ott, Johannes Ring, Franziska Ruëff, Bernhardt Sachs, Helmut Sitter, Bettina Wedi, Stefan Wöhrl, Margitta Worm, Torsten Zuberbier

**Affiliations:** 1 Department of Dermatology and Allergology Biederstein, Faculty of Medicine, Technical University of Munich, Munich,; 2 Department of Dermatology and Allergology, Germany, Aachen Comprehensive Allergy Center (ACAC), University Hospital of RWTH Aachen University, Aachen,; 3 Department of Dermatology and Allergology, Allergy Center Mainfranken, University Hospital Würzburg, Würzburg,; 4 Department of Dermatology and Allergology, University Hospital Giessen and Marburg, Marburg,; 5 Department of Dermatology, Venerology and Allergology, University of Leipzig, Leipzig, Germany,; 6 Facoltà di Scienze biomediche, Università della Svizzera italiana, Lugano, and Department of Dermatology and Allergology, University Hospital Basel, Switzerland,; 7 Department of Dermatology, Münster University Hospital, Münster,; 8 Department of Dermatology, Venereology and Allergology, University Medical Center Göttingen, Göttingen,; 9 Department of Dermatology, Venerology and Allergology, St. Josef Hospital, University Hospital of the Ruhr University Bochum, Bochum,; 10 Department of Dermatology and Allergology, University Hospital, Justus-Liebig University, Gießen,; 11 Department of Dermatology and Venereology, University Hospital Halle, Halle (Saale),; 12 Pediatric Clinic, Marienhospital Bonn, Bonn,; 13 Documentation Center for Severe Skin Reactions, Department of Dermatology and Venereology, University Medical Center Freiburg, Freiburg,; 14 Asthma Allergy Center Leverkusen, Leverkusen,; 15 Children’s and Youth Hospital Auf der Bult, Hanover,; 16 Department of Dermatology and Allergology, Allergy Center, Ludwig Maximilian University of Munich,; 17 Institute for Theoretical Surgery, Philipps University of Marburg, Marburg,; 18 Hanover Medical School, Department of Dermatology, Allergology and Venereology, Hanover, Germany,; 19 Floridsdorf Allergy Center (FAZ), Vienna, Austria, and; 20 Allergology and Immunology, Department of Dermatology, Venereology and Allergology, Charité-Universitätsmedizin Berlin, Berlin, Germany

**Keywords:** drug hypersensitivity, allergy diagnostics, history, skin test, in vitro examination, provocation test

## Abstract

Not available.

## What’s new? 

The basic principles for diagnosing allergic and non-allergic hypersensitivity reactions to drugs have not changed since the last version of this guideline dated December 31, 2014. The guideline was adapted or supplemented accordingly during the current review in view of new publications on the significance and informative value of specific test procedures for drug hypersensitivity. 

## The most important recommendations at a glance 

Drug hypersensitivity reactions presenting as urticaria/anaphylaxis within 1 (– 6) hours after drug administration (“immediate reactions”) or as exanthem several hours to days later (“delayed reactions”) should be clarified allergologically (strong consensus). Appropriate allergological diagnostics should be used to try to prove the involvement of the immune system (allergy) (strong consensus). The clinical symptoms of an allergic reaction are usually much more severe than those of a non-allergic intolerance reaction. We recommend allergological diagnostics on the one hand to identify patients at risk unambiguously in a timely manner, but on the other hand also to prevent unjustified restrictions in drug therapy. Allergy diagnostics should be performed within 4 weeks to 6 months after the reaction (strong consensus). The classification of a drug reaction and thus the planning of allergy diagnostics should be based on the clinical picture, the time course (chronology), and the suspected causative drug (strong consensus). If the result of a validated skin and/or laboratory test is clearly positive in conjunction with a matching medical history, the trigger(s) can be considered sufficiently confirmed (strong consensus). If no diagnosis is possible after the skin and laboratory diagnostics, controlled provocation testing should be sought after a risk-benefit assessment (strong consensus). The result of the allergological diagnosis should be explained in detail to the patient (strong consensus). A diagnosed allergic or non-allergic hypersensitivity to one or more drugs should be documented in a drug allergy passport to ensure that this drug(s) will be avoided in the future; in individual cases, if known and considered useful, tolerated alternative drugs can also be mentioned in the allergy passport (strong consensus). 

## External appraisal and adoption 

The guideline was approved by the boards of all participating professional societies from December 2022 to April 2023. 

## Validity period and updating procedure 

The guideline is valid from July 1, 2023 until the next update; the validity period is estimated to be 5 years. Regular updates are planned; in case of urgent need for changes, these will be published separately. Comments and advice for the update process are explicitly welcome and can be sent to the Guideline Secretariat (contact: see correspondence address). 

## Recommendations and consensus 

In the manuscript, a strong recommendation is indicated by the phrase “we recommend” and a conditional or weakened recommendation is indicated by “we suggest”. An open recommendation is marked by “may”. Consensus strength was defined as follows: strong consensus > 95%, consensus > 75 – 95%, majority agreement > 50 – 75%, no agreement < 50%.[Table Abbreviations]

## Introduction 

While type A adverse drug reactions (ADRs) are caused by a known pharmacological-toxic mechanism (“on-target reactions”), allergic and non-allergic hypersensitivity reactions to drugs are based on an individual disposition of the affected patients and are in principle unpredictable (type B, “off-target reactions”) [1, 2]. In this context, drug allergy must be distinguished from a non-allergic (non-immunological) drug hypersensitivity reaction (Table 1). 

We recommend to clarify any suspected hypersensitivity reaction in connection with the use of medical products with the aim of identifying the trigger and, if necessary, the mechanism, assessing the risk of subsequent reactions for patients, and counseling patients in this regard (strong consensus) [2]. Failure to carry out diagnostic workup may result in a severe reaction after the drug has been taken or administered again, and may also lead to an unjustified restriction of drug therapy. 

The Working Group on Drug Allergy was commissioned by the DGAKI to update the guidelines published in 2008 and 2015 [2, 3]. KB, WP, HO, AT, GW, and RT initially prepared a preliminary version by updating the literature and revising the existing guideline. Together with experts from other professional societies and institutions with special experience in the care of patients with hypersensitivity reactions to drugs, recommendations were revised and newly created based on current literature, clinical experience of participants, and theoretical considerations. In consensus conferences on November 5, 2021 and September 28, 2022, each of these recommendations was discussed and agreed upon in a structured consensus-building process under neutral moderation by an external neutral moderator, where contentious issues were clarified and a final formulation was found. 

Up to 10% of the general population report a history of a hypersensitivity reaction to drugs. This impressive number contrasts with a small number of hospital departments and practices specializing in drug reactions so that a significant undersupply can be assumed in this area of our healthcare system. Moreover, only a fraction of suspected hypersensitivity reactions to drugs can actually be confirmed by allergy diagnostics, including controlled provocation, for example, after presumed reaction to a penicillin in less than 10% of cases [4]. Thus, in most cases, allergological workup can exclude drug hypersensitivity so that the medically and economically optimal drug will be available to the affected person again in the future. 

The primary aim of this guideline is to improve the quality of allergy care by explaining the general principles of diagnosis of hypersensitivity reactions to drugs, highlighting new developments, and identifying deficits and controversies. It adresses all physicians and other healthcare professionals involved in the diagnosis and counseling of patients of all ages with a (suspected or confirmed) hypersensitivity reaction to one or more drugs. Regarding methodological details on diagnostic procedures, reference is made to relevant position papers [5, 6, 7, 8, 9, 10, 11, 12, 13, 14, 15, 16, 17, 18, 19, 20, 21]. This guideline also does not address certain very rare clinical pictures that may be triggered by drugs [22]. 

## Definition and classification 

The most important definitions and the classification of drug reactions commonly used today are summarized in Table 1 [1, 2, 23]. We recommend to perform allergy diagnostics only for hypersensitivity reactions (type B, “off-target reactions”), not for the pharmacological toxic drug side effects (type A, “on-target reactions”) (consensus). Whereas a non-allergic immediate reaction is usually less severe and often largely confined to the skin with flushing and urticaria, an allergic, IgE-mediated immediate reaction often results in a moderate to severe anaphylactic reaction. 

An initial classification based on clinical presentation, chronology/time course of the reaction, and the suspected trigger determines the selection of planned diagnostic procedures (Table 2) [1, 24]. 

## Diagnosis 

We recommend that diagnostics are put in the hands of a physician or allergology center with experience in allergology (consensus). Knowledge of drugs that frequently elicit specific hypersensitivity reactions is essential for diagnostic planning in order to be able to assess the probability with which a drug has caused a reaction. History, skin, in vitro, and provocation tests are available to identify a drug that has triggered a hypersensitivity reaction. Of utmost importance is the most accurate description and classification of the suspected clinical reaction. The diagnostic procedure must take into account the particular features of the individual case and the diagnostic possibilities. 

If possible, allergological workup should be sought within 4 weeks to 6 months after the symptoms have resolved. There are indications that the detection of hypersensitivity is less successful with increasing time interval to the suspected clinical reaction [25, 26]. We explicitly do not recommend (among other things, due to the (theoretical) risk of iatrogenic sensitization) allergological workup in patients with no previous history of a drug hypersensitivity reaction (so-called “prophetic testing”) [27]. 

## Medical history and clinical manifestations 

Pre-test probability can be improved if the patient is already seen by the allergist or dermatologist in the acute phase of a suspected drug reaction, as the latter can then more easily delineate differential diagnoses, classify the clinical picture, assess the course of the symptoms and better assess the possibility of a connection with the intake of a drug. Photo documentation of the skin changes in the acute phase can be very helpful in classifying the clinical reaction. If multiple drugs are administered, we suggest to create a timeline diagram (strong consensus) [22]. In this case, the time interval between the first contact with the accused drug and the appearance of skin lesions may already indicate a possible pathomechanism (Table 3) [28]. We suggest that the anamnestic data and all available medical records concerning the reaction (e.g., physician’s letter, medical chart, anesthesia protocol) are used to classify a reaction (Table 2) (strong consensus). A standardized questionnaire, which was published some time ago, can help to actually capture all significant events and circumstances in the patient’s history [13, 29]. This information can then be used to draw up an appropriate test plan (Table 4). 

## Skin tests 

Skin tests are indicated to detect sensitization in cases of hypersensitivity reactions with symptoms of an allergic reaction [18, 30]. With the exception of two penicillin test solutions (which contain benzylpenicilloyl and benzylpenilloate and are therefore only suitable for detecting sensitization to benzyl penicillin/penicillin G), no drug solutions approved under pharmaceutical law are available for skin testing in Germany up to July 2023. So far, there is no generally accepted standard for skin tests with medicinal products. We suggest to follow the methods of the European Network on Drug Allergy (ENDA) and that the existing drug- and method-specific guidelines are applied (strong consensus) [10, 17, 30]. For special drugs with which a provocation test seems hardly practicable, e.g., for muscle relaxants or injection narcotics, the skin test is the only available diagnostic method besides in vitro studies [10, 31, 32]. 

However, positive, diagnostically meaningful skin test reactions occur in only some of the patients with allergic hypersensitivity reactions. If drugs are tested in too high concentrations, irritative (false-positive) test reactions are possible. [17]. We suggest to use non-irritative test concentrations if known (consensus). Recommendations have been made for a number of drugs; examples are listed in Table 5 [17]. Natural rubber latex or antiseptic allergy should be ruled out in cases of immediate reactions, if appropriate. 

In very rare cases, the skin test (the intradermal test, less so the skin prick test) with the causative drug can trigger a hypersensitivity reaction, and even a severe systemic reaction. Therefore, the medical and other healthcare professionals responsible for the testing should be prepared for such emergency situations [30, 33, 34, 35]. Skin testing with non-standardized drug solutions requires special care; stepwise testing with gradually increasing concentrations of the test substance, e.g., at 30-minute intervals (first 1 : 1,000, then 1 : 100, and only then 1 : 10) can greatly reduce the risk of an anaphylactic reaction in the context of an intradermal test [30]. 

Re-sensitization by the skin test itself is (theoretically) possible, the risk depends on the substance tested, the concentration, and the test method. We suggest that skin tests should only be performed with the presumed reaction-causing drug, potentially cross-reacting molecules, or alternatives to be used (consensus). 

### Test material

- Medical preparations (ideally the exact preparations used), the active substance, sometimes excipients (hypersensitivity reactions to excipients are rare and need to be considered in individual cases).
- Positive and negative controls depending on the test procedure
- Appropriate test concentration to avoid irritative or pharmacological reactions (e.g., test for morphine derivatives, quinolone antibiotics) or false-negative test reactions (threshold test, if applicable).
- Suitable preparation of the material for the skin test (an intradermal test, for example, is only possible with a sterile drug solution).
- The significance of skin tests for the detection of sensitization varies considerably for different drugs. For some drugs, the diagnostic sensitivity of skin tests could either not be confirmed at all or only in a few individual cases, the classic example being the large group of non-steroidal anti-inflammatory drugs (NSAIDs) with the exception of, e.g., metamizole [17].

### Test procedure

- Sufficient time interval from drug reaction and administration of antiallergic medication.
- If there is a risk of triggering a systemic reaction, medical monitoring of the patients over a sufficiently long period of time, if necessary threshold test with diluted solutions.
- The skin prick test is less sensitive compared to the intradermal test, but also less risky [17]. In case of immediate reactions, we recommend to perform a skin prick test first (strong consensus). An intradermal test should be performed if no meaningful result can be obtained by the skin prick test (strong consensus). For individual drug groups, e.g., local anesthetics, a direct intradermal test may be justifiable (strong consensus). If a late reaction is suspected, we recommend to do a patch test (open before closed before strip-off patch test, if necessary) and/or an intradermal test with late reading (strong consensus) [36]. If photo-induced reactions are suspected, additional tests in combination with UV irradiation (e.g., photo patch test) should be used (strong consensus).
- In children, we recommend to narrrow down intradermal testing to most important preparations, especially in infants and toddlers (strong consensus) [21].
- The timing (simultaneous or consecutive diagnosis of different substances or substance concentrations) is based on the suspected pathomechanism, the severity of the reaction, and the risk of the chosen skin testing methodology (consensus). The time intervals between threshold tests should be based on the initial reaction, i.e., these may be 20 minutes for immediate-type reactions and 2 days for late-type reactions.
- We suggest to take the reading of the test reaction after 15 – 20 minutes for the skin prick or intradermal test, and after (24 or) 48 and at least 72 hours for the patch test (consensus). In the case of exanthem, we recommend a late reading to be taken with the skin prick and intradermal test (example: clarification of an amoxicillin exanthem) (strong consensus). In case of anaphylactic symptoms and higher test risk, an open patch test with early reading after 20 – -30 minutes can be performed (example: clarification of anaphylaxis after topical application of bacitracin) (strong consensus). Note: Skin test reactions, especially with certain drugs (e.g., glucocorticoids), can also occur at a later time, sometimes even after > 1 week. Since skin test reactions can also occur after several days, we suggest to advise patients developing a positive reaction at a later time point to present again at the allergy department for an additional reading (strong consensus).
- After a fixed drug reaction, a skin test directly in the area of a previously affected skin site, a so-called intradermal test in loco or a patch test in loco, can increase the sensitivity of the diagnosis.

### Evaluation

- Reading according to the criteria of the applied test procedure and documentation of morphological peculiarities.
- In the case of reactions to medicinal preparations, further diagnostic workup with individual ingredients may be useful in individual cases, if available.
- In case of skin test reaction, a non-specific reaction is to be excluded as far as possible. For this purpose, data from the literature on non-irritant test concentrations are used [17].
- Only when non-irritative test concentrations are used is it sometimes possible to make a definitive diagnosis of allergy with a positive skin test (sensitization) in connection with the medical history (e.g., in the case of beta-lactam antibiotic or heparin allergy) [4, 6]. In other cases, further investigations (in vitro diagnosis, provocation tests) are necessary.
- In cases of unclear skin test reactions, we suggest to perform additional skin tests with higher dilution levels to exclude irritative test results in order to better assess the likelihood of a specific reaction (consensus) [17].

## In vitro diagnosis 

Laboratory testing can be particularly helpful in cases of negative skin tests as well as severe drug reactions, especially when a provocation test cannot be performed or the skin test itself could pose a potential hazard, such as after an anaphylactic reaction to a beta-lactam antibiotic [4, 6]. 

### In vitro diagnosis with drugs 

Numerous laboratories offer the determination of specific IgE antibodies against various drugs [14, 37]. Cellular assays are also sold commercially, but the evaluation of the measurement results requires special experience with these methods; they also require prompt processing of blood samples and a larger blood volume, which can cause problems, especially in children [38, 39].

- Validated tests for the detection of specific IgE antibodies in serum are only available for a few drugs (Table 6) (mainly beta-lactam antibiotics), otherwise no standardized and evaluated in vitro methods exist [4]. We suggest against using non-validated IgE assays to drugs (strong consensus).
- The validity of the detection of specific IgE antibodies to drugs remains poorly established.
- Other immunological laboratory methods (e.g., basophil activation test, basophil histamine release test, leukotriene release test (CAST), lymphocyte transformation test, lymphocyte activation test, ELISpot test) cannot be regularly used in standard clinical diagnosis because they are only available in a few laboratories. In many cases, the methods are not standardized and the few data on diagnostic sensitivity as well as specificity with regard to individual drugs are not sufficiently validated. Laboratory results must be evaluated cautiously and considered in conjunction with all other findings. However, we suggest that they can be an important complementary diagnostic component for centers with sufficient experience (consensus) [40, 41, 42, 43, 44].
- Reliable detection or exclusion of drug hypersensitivity based on in vitro diagnosis alone is not possible. We recommend to interpret in vitro test results in the context of history/clinical manifestation and, wherever possible, in vivo testing (strong consensus) [14].

### Other in vitro studies

- If clinical symptoms are appropriate, drug-metabolizing enzymes may be determined to detect metabolic disorders associated with hypersensitivity to certain drugs (e.g., thiopurine S-methyltransferase (azathioprine)) (consensus).
- Prior to the administration of abacavir, pharmacogenetic testing for HLA B*5701 expression is recommended in its summary of product characteristics [45, 46]. Such a procedure is also advisable before prescribing carbamazepine for patients of Southeast Asian origin, in which case HLA B*1502 is relevant [46].
- In patients of European origin, the allele HLA B*5701 has been identified in association with carbamazepine-induced severe skin reactions, but the association is not as strong as that observed in Southeast Asians. In allopurinol-induced severe skin reactions, an association of HLA B*5801 and severe skin reactions has been observed for Europeans as well as for Asians, but there is no recommendation in the summary of product characteristics for an appropriate pharmacogenetic study, although this could help to reduce the number of severe skin reactions triggered by allopurinol.
- In cases of questionable anaphylaxis, the increase in mast cell mediators (especially tryptase) may provide evidence that mast cell activation has occurred. We suggest blood draws to be preferably taken ~ 1 – 2 hours after the onset of a reaction and 2 – 3 days after the reaction has resolved (baseline tryptase level) (consensus). An increase in the post-reaction tryptase value of 2 ng/mL + 20% of the basal value is used as an indication of mast cell activation [47, 48].

## Provocation tests 

We recommend to perform provocation tests when the trigger of drug hypersensitivity cannot be identified with reasonable certainty by history, skin testing, and in vitro studies and when the benefit exceeds the risk (strong consensus) [15]. Because of the limited power of other diagnostic methods, the provocation test is still considered the gold standard in the workup of a suspected allergic or non-allergic hypersensitivity reaction to drugs. Especially in cases of suspected intolerance to important and therefore permanently hardly dispensable drugs or drug groups, e.g., NSAIDs, certain antibiotics, or local anesthetics, provocation tests often serve primarily to prove tolerance. In individual cases, in the event of a possible cross-reaction, the tolerability of an alternative (fallback) drug can also be tested in a provocation test [6, 49]. Indications for drug provocation tests are [15]:

- Exclusion of hypersensitivity in case of unclear history.
- Confirmation of the diagnosis in case of suspicious history with negative, not convincing, or not available other allergy diagnostics.
- Exclusion of a cross-reaction with chemically/structurally similar drugs (active ingredients).

The effort involved in the clarification of drug intolerance reactions is often underestimated outside the field of allergology. In order to enable the diagnosis of as many patients as possible with limited resources, it is therefore essential for a specialized clinic department or practice to prioritize according to the importance of the drugs to be clarified:

- Highest importance: urgently needed drugs taken by patients without direct medical supervision (antibiotics with a broad spectrum of activity such as beta-lactams, painkillers), individually essential drugs (e.g., insulin for diabetics, antiepileptics, neuroleptics).
- High importance: other important drugs that will probably have to be used in the future (e.g., antibiotics with narrow spectrum of activity, e.g., clindamycin; tetracyclines; analgesics as alternative drugs; X-ray contrast media, local anesthetics).
- Moderate importance: drugs for which an acceptable substitute already exists.
- Low importance: Non-essential medicines such as vitamins and supplements.

We recommend to inform the test person or their legal guardian about the aim of the diagnosis, the risk, alternatives, and the test procedure including the use of placebo. Consent for provocation tests should be given in writing (consensus). 

A medically supervised follow-up period should be maintained after provocation as long as severe reactions (e.g., anaphylaxis) are expected (consensus). Therefore, provocation tests where severe reactions are expected should be performed under inpatient conditions with availability of immediate emergency care (experienced medical and nursing staff, appropriate drug and device equipment) (strong consensus). 

We recommend that the determination of the procedure for drug provocation testing always remains a case-by-case medical decision in which numerous factors and patient-specific characteristics (e.g., type of drug, estimated likelihood of reaction, expected severity of reaction, patient’s expectation/fear) must be considered (strong consensus). 

The basis of provocation tests is to apply the test substances in the form in which they led to the hypersensitivity reaction. We recommend that also oral provocation may be performed if the drug was administered by another route (e.g., intramuscularly, intravenously, or rectally) when the suspected clinical reaction occurred (strong consensus). 

Depending on patient-specific characteristics, we recommend to complement provocation tests with placebo tests, as certain reactions may also occur after administration of a placebo (strong consensus). 

### Test material

- Drug product, individual active ingredients if necessary; excipients only in individual cases; in the case of combination preparations, if necessary, drug product identical in composition/manufacturer to that of the original reaction.
- Preparation of the test material in a form suitable for single/double-blind and fractionated delivery.
- When clarifying reactions to some drugs (e.g., NSAIDs), it may be appropriate to also check alternative drugs as part of the provocation test.

### Test procedure

- Sufficient time interval (after an immediate reaction ~ 2 weeks; after a late reaction ~ 4 – 6 weeks; if no urgent need, e.g., surgery currently necessary) to the drug reaction and to anti-allergic medication.
- For provocation tests with the possibility of triggering systemic reactions, in-hospital monitoring of patients required.
- Adequate monitoring for the entire duration of the provocation test and a safety interval depending on the reaction type after administration of the last test dose.
- We recommend that medications and other equipment for emergency treatment is always available; staff should be familiar with treating acute emergencies (strong consensus).
- Consideration of the pharmacological effects of drugs (e.g., narcotics, antidiabetics, neuroleptics, heparins) and the respective maximum doses as well as any altered pharmacokinetics on the part of the patient (e.g., liver, kidney dysfunction).
- Administration of the drug in gradually increasing doses when administered systemically (e.g., (1%) – 10% – 50% – 100% or (1%) – (3%) – 10% – 30% – 100% of the usual single dose, if necessary up to the daily dose or anamnestically applied dose) with a time interval depending on the suspected reaction mechanism (30 minutes for immediate reaction to 2 days for late reaction); if necessary continued administration in therapeutic daily dose for several days, e.g., in case of drug exanthem).
- If a summation reaction is suspected, e.g., drug-dependent, exertion-induced anaphylaxis, the additional trigger factors, in this example physical exertion, should be considered in the provocation testing.
- Single-blind (double-blind) tests with appropriate placebo controls.
- The results of non-blinded provocation tests can only be used diagnostically in the case of a negative test result or clear clinical symptoms.
- The placebo controls should be given in multiple administrations just as the verum controls.
- Treatment of any dangerous test reactions that occur.
- Due to a possible refractory phase, a sufficiently long time interval must be allowed between a positive test reaction and further follow-up tests.
- Informing patients about what to do if a reaction occurs after the end of medical monitoring.

### Evaluation 

We recommend to evaluate the results of provocation tests using objectifiable parameters; subjective symptoms may also be included in the evaluation (strong consensus).

- Documentation of symptoms and temporal development of the reaction, as far as possible, recording of objectively measurable parameters, e.g., blood pressure, FEV_1_, serum tryptase.
- In the absence of a reaction to a preparation of the suspected drug (active ingredient) prepared by a pharmacy, additional provocation with the drug preparation used by the patient may be useful.
- After a particular drug has been tolerated in a provocation test, it can never be ruled out that a new hypersensitivity or sensitization to precisely this drug will develop later. In addition, a negative provocation test cannot provide 100% certainty that the tested drug will actually be tolerated later in the therapeutic dose. In particular, the influence of co-factors such as a concomitant disease, e.g., a viral infection, drug interactions or an increase in reactivity may be responsible for a “false negative” result.
- The predictive value of a negative provocation with drugs was nevertheless regularly > 95% in studies. The clinical symptoms of a renewed hypersensitivity reaction after a preceding negative provocation test are usually minor [50, 51].

In the case of long-standing severe immediate reactions and a one-time inconspicuous provocation test, a re-evaluation (repetition of skin and in vitro diagnostics, if inconspicuous also provocation tests) can be performed in the case of high suspicion [52] (consensus). 

### Contraindications 

The contraindications mentioned below refer to the provocation test with the suspected drug as well as a possibly cross-reacting drug (due to chemical structural similarity):

- Pregnancy and lactation.
- Risk of very severe hypersensitivity reaction, e.g., Stevens-Johnson syndrome (SJS), toxic epidermal necrolysis (TEN), drug reaction with eosinophilia and systemic symptoms (DRESS), or drug-induced hepatitis.
- Concomitant diseases that could aggravate a hypersensitivity reaction or complicate its treatment, such as uncontrolled asthma, severe chronic obstructive pulmonary disease (COPD), coronary heart disease, etc.
- Insufficient compliance, lack of patient understanding of the procedure, lack of informed consent.
- Taking drugs that might suppress or mask a positive response, e.g., H_1_ antihistamines, immunosuppressants including systemic glucocorticosteroids in long-term use > 10 mg prednisolone equivalent per day or short-term use in higher doses (> 3 days > 50 mg/day and > 7 days > 30 mg/day) [18].

In individual cases, a provocation test with a therapeutically urgently needed drug may be justified despite a contraindication (e.g., intake of antihistamines or glucocorticosteroids if these cannot be discontinued and had been taken in parallel to the suspected trigger). Careful consideration of the benefit of provocation testing and the risk of a (possibly severe) positive reaction is essential in each individual case. 

## Overall assessment 

**Figure 1.**
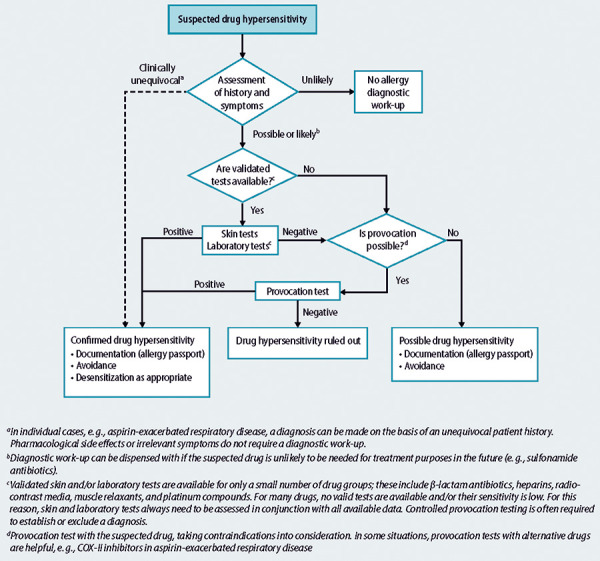
Diagnostic algorithm for suspected drug hypersensitivity [2].

The allergological diagnosis should be made from a synopsis of the medical history and the findings of skin, in vitro, and provocation tests (Figure 1). In addition, the probable mechanism of an allergic or non-allergic intolerance reaction and a possible cross-reaction between chemically/structurally similar drugs should be evaluated. 

A definite exclusion of hypersensitivity to a drug is not always possible even when all available test methods are used. In many cases, however, the allergist can assess the likelihood of hypersensitivity or sensitization, a possible cross-reaction, and a severe allergic reaction on the basis of the patient‘s medical history and the results of the diagnostic tests and, if necessary, point out alternative medications in the allergy passport. 

We recommend to document the result of the overall assessment and to discuss it with the patient (strong consensus). 

In addition, an allergy passport must be issued, the contents of which must be strictly observed in the patient‘s future drug therapy [19]. We recommend to name the clinical manifestations and the triggers (international non-proprietary name (INN), drug, if applicable) in the allergy passport; there should be an indication of possible cross-reactions (strong consensus). 

We suggest to indicate possible (tested) alternative substances/preparations with the maximum tolerated single and cumulative daily dose (consensus). 

Notes on possible pharmacoprophylaxis of hypersensitivity reactions (e.g., premedication for administration of radiographic contrast media or for surgical procedures under general anesthesia) may be added (strong consensus). 

We recommend that allergy passports are only issued by physicians experienced in allergology (strong consensus). Medications tolerated by the patient based on medical history can be entered in the allergy passport by the physician with a corresponding note. Entries by patients themselves are not permitted. 

## Diagnostics for special patient groups 

### Children 

In principle, children can be tested using the same methods as adults, although most testing procedures are less well studied in children. However, because of the painfulness, intradermal tests are used more reluctantly. Diagnostic workup of a questionable hypersensitivity reaction to a drug is often particularly important in childhood because the choice of alternative drugs to treat a specific condition, e.g., antibiotics or NSAIDs, is often significantly limited compared with adults [21]. 

Children more frequently develop an infection-associated exanthem in the context of febrile infections, which is not infrequently misinterpreted as a drug reaction because NSAIDs or antibiotics were administered at the same time for therapy. On the other hand, a mild maculopapular exanthem (MPE) may also be an expression of an allergic reaction to a drug. However, drug hypersensitivity in children is very rare compared to adults, and therefore an oral provocation test can be performed directly without prior skin testing to clarify a MPE (“benign rash”) [53]. The significantly lower effort facilitates the “de-labeling” of a putative drug hypersensitivity. 

Even in the case of proven sensitization, spontaneous tolerance development seems to be possible over the course of years [54]. Therefore, even in the case of proven sensitization or allergy, a new diagnosis including provocation may be useful after several years. 

### Pregnant women 

Allergological in vivo testing can be considered in pregnancy at most in case of urgent indication, e.g., a drug absolutely needed for delivery, after consultation with the attending gynecologist and careful consideration of benefits and possible risks. 

### Elderly patients 

For elderly patients, certain cardiovascular or pulmonary pre-existing conditions may be a risk factor for a more severe and more difficult-to-treat reaction in the provocation test. In addition, it is often difficult to distinguish a hypersensitivity reaction from the underlying disease, since, for example, circulatory symptoms and respiratory distress can occur both in the context of anaphylaxis and be caused by the underlying disease. 

### Chronically ill patients 

A serious chronic disease itself and its treatment must be taken into account when planning the allergological diagnosis of a hypersensitivity reaction to drugs and may speak against provocation tests as a relative contraindication. In the dosage of drugs, hepatic or renal insufficiency as well as drug interactions when taking numerous drugs must be taken into account, which may result in overdose or underdose. A persistent inflammatory reaction, e.g., due to certain autoimmune diseases or a viral infection with HIV, HCV, or human herpes viruses can increase the probability of a hypersensitivity reaction to a drug due to a persistent stimulation of immune cells (“danger signal”). 

Long-term immunosuppression with medication, e.g., in organ transplant recipients or rheumatological patients, can severely limit the validity of skin tests. In the case of chronic urticaria with accompanying urticarial dermographism, both skin tests and provocation tests are hardly practicable and difficult to assess. 

## Controversies and deficits in the diagnosis of drug hypersensitivity reactions

- Today, almost 10% of the German population report a penicillin allergy in their medical history. The vast majority of these supposed hypersensitivity reactions are never verified, a bacterial infectious disease of the affected persons is therefore usually treated with expensive and for various reasons disadvantageous reserve antibiotics. In view of the large number of patients, a complex diagnosis by an allergy specialist including skin test, in vitro test, and subsequent provocation is hardly possible. In the meantime, several publications show that certain cases with an apparently very low risk of hypersensitivity reactions do not necessarily need to be tested [55, 56, 57]. The current studies on the assessment of the probability regarding drug hypersensitivity by means of a standardized questionnaire or algorithm are therefore very welcome, in the hope that they will allow direct “de-labeling” in many cases even without testing.
- Following a mild MPE (benign rash), direct oral provocation without prior skin testing has been suggested by some research groups, as is already recommended for children with such a mild reaction associated with infection and antibiotic use.
- The effects of a not clearly confirmed drug hypersensitivity on the quality of life of those affected have not been adequately studied. In many cases, anxiety disorders develop in connection with the use of drugs. A diagnosis that either confirms or reliably rules out drug hypersensitivity can have a positive effect on the quality of life in addition to facilitating future drug treatment.
- Testing options for non-allergic drug reactions have been limited to history/clinical symptoms and provocation testing. A better understanding of the underlying mechanism, e.g., on the importance of the Mas-related G-protein-coupled receptor X2 (MRGPRX2), could allow the development of new test methods.
- Better test solutions and thus an improved sensitivity of the diagnostics is conceivable by the targeted use of allergenic determinants, of metabolites, or drug-carrier conjugates.
- The sensitivity and specificity of skin tests and in vitro tests are mainly dependent on the type of drug and the clinical reaction form. Meaningful data are available so far only for a few drugs or drug groups, which should be supplemented and extended by future studies.
- Compared to acute generalized exanthematous pustulosis (AGEP) and drug reactions with eosinophilia and systemic symptoms, the sensitivity in the SJS-TEN spectrum can be considered very low (< 25%) [58, 59, 60].
- Data on cross-reactivity between different beta-lactam antibiotics have so far been based mainly on skin test results. Further studies are desirable on this question as well as on cross-reaction within other drug groups, e.g., X-ray contrast media or fluoroquinolones.
- In vitro diagnosis for drug hypersensitivity is not yet a routine method, with the exception of IgE against certain beta-lactam antibiotics. Laboratory test methods need to be further developed and investigated – possibly also with targeted use of crucial drug metabolites – so that in the future they may possibly allow the diagnosis or definite exclusion of drug hypersensitivity even without provocation testing. By measuring pharmacogenetic characteristics (e.g., polymorphism of HLA or metabolizing enzymes) even before the drug is administered, certain hypersensitivity reactions can already be prevented.
- Studies comparing the validity of allergy diagnosis with medications in children versus adults would be desirable, because currently it is primarily adult experience that is extrapolated to pediatric populations.
- Regarding the duration of the provocation test with drugs, there is no generally accepted standard so far – even for common reaction forms such as MPE. Most guidelines consider provocation with a daily dose of the suspected drug administered in gradually increasing amounts within 1 day to be sufficient, while individual centers recommend longer-term administration for up to 7 days [15, 61].
- For skin testing with drugs, with the exception of two benzyl penicillin solutions with a narrowly limited indication, no test solutions have been approved under drug law and are commercially available to date. Allergists are therefore forced to resort to solutions that they prepare themselves from finished medicinal products. On the one hand, this makes diagnosis time-consuming, complicated, and error-prone; on the other hand, certain legal requirements must be observed, which can unsettle both the staff involved and the patients. Due to the use of ready-to-use drugs, diagnosis is often expensive, complex, complicated, and error-prone with regard to the preparation of the test. Manufacturers are withdrawing more and more parenterally available drugs from the market. This further limits the performance of intradermal testing. Approved test solutions for the most important drugs or drug groups would be desirable, but are not to be expected in view of the great effort and at the same time low economic benefit for the manufacturer.
- Due to the current lack of cost-covering reimbursement in the German healthcare system, allergological diagnosis of drug hypersensitivity reactions is currently only offered in a few specialized hospital departments and specialized practices. The omission of diagnostic workup in cases of suspected drug hypersensitivity can lead both to a renewed clinical reaction, because the causative drug was not identified, and to an unjustified restriction of drug therapy, because an alleged hypersensitivity could not be excluded. In particular, the authors believe that appropriate reimbursement for allergological diagnostics is urgently needed to improve the care of patients with drug hypersensitivity in the future.

## Funding 

The costs of the consensus conferences were financed by the lead professional society DGAKI. No further costs were incurred. 

## Conflict of interest 

The information on interests was collected using the AWMF form of 2018 and assessed by Prof. Dr. Monika Raulf for a thematic relation to the guideline. Guideline-relevant lecturing or consulting activities for manufacturers of devices for allergy diagnosis were categorized as a low conflict of interest, and direct financial secondary interests (e.g., share ownership, patent) as a moderate/high conflict of interest. Low conflicts of interest resulted in abstention from voting. Strong conflicts of interest were not present. Protective factors counteracting conflict of interest bias were the pluralistic composition of the guideline group, structured consensus building under neutral moderation, discussion on interests, and handling of conflicts of interest at the beginning of each consensus meeting, and a public consultation version. 

**Abbreviations. Abbreviations:** Abbreviations.

ADR	Adverse drug reaction
AGEP	Acute generalized exanthematous pustulosis
AWMF	German Association of the Scientific Medical Societies
COPD	Chronic obstructive pulmonary disease
DGAKI	German Society for Allergology and Clinical Immunology
DRESS	Drug reaction with eosinophilia and systemic symptoms
INN	International non-proprietary name
MPE	Maculopapular exanthem
NSAID	Non-steroidal anti-inflammatory drugs
SJS	Stevens-Johnson syndrome
TEN	Toxic epidermal necrolysis

**Table 1.** Definitions.

**Adverse drug reaction (ADR):** A noxious and unintended reaction that occurs in addition to the intended effect of a drug, for which a causal relationship between the use of the drug and the adverse effect is suspected. ADRs can be both type A (pharmacological/toxic) and type B (hypersensitivity).
**Type A (“augmented” = pharmacological/toxic (on-target) drug effects)**: Disease manifestations due to dose-dependent predictable pharmacological/toxic effects of a drug at the recommended dose (examples: sedative effect of older antihistamines, hair loss due to cytostatics) or increased dosage (intoxication).
**Type B (“bizarre” = hypersensitivity (off-target) reactions)**: Individual, unpredictable clinical reaction to a drug, i.e., symptoms occur only in specially predisposed patients. Two forms can be distinguished:
–**Drug allergy**: Hypersensitivity is based on an immunological reaction (types I – IV according to Coombs and Gell).
–**Non-allergic drug hypersensitivity**: An allergic mechanism is not detectable. Previously, this type of reaction was further divided into:
–**Drug intolerance**: Typical symptoms of pharmacological action (toxicity) develop even at low doses that are usually tolerated.
–**Drug idiosyncrasy:** The symptoms differ from the pharmacological substance effect. In cases where the symptomatology of this form of hypersensitivity looked similar to an allergic reaction, the term pseudoallergy has also been used.

**Table 2.** Time interval, clinic, and pathomechanism – three levels for classifying a drug hypersensitivity reaction.

**1) Time reaction intervals**
a) For those already sensitized
–Immediate reaction (“immediate”) immediate up to 60 minutes (in rare exceptions up to 6 hours)
–Delayed reaction (“non-immediate”) > 6 hours to several weeks
b) In case of new sensitization under therapy
–Typical sensitization latency 7 – 10 days
**2) Clinical manifestations**
a) Immediate reaction: E.g., flush, urticaria, angioedema, bronchospasm, anaphylaxis.
b) Late reaction: Maculopapular exanthem (MPE), acute generalized exanthematous pustulosis (AGEP). Severe cutaneous drug reactions: Stevens-Johnson syndrome (SJS), toxic epidermal necrolysis (TEN), drug reaction with eosinophilia and systemic symptoms (DRESS).
c) Others: E.g., hepatitis, cytopenia, autoimmune diseases (e.g., lupus erythematosus, drug-induced linear IgA dermatosis)
**3) Pathomechanism**
a) Allergic hypersensitivity: Immediate type (type I according to Coombs and Gell, IgE mediated): typical manifestation, immediate-type symptoms (reaction time: 0 – 6 hours)
b) Non-allergic hypersensitivity: Typical manifestation immediate-type symptoms (reaction time: 0 – 6 hours, rarely up to 12 hours)
c) Allergic hypersensitivity: Delayed-type (type IV according to Coombs and Gell, T-cell mediated): typical manifestation delayed-type (reaction time: 24 – 72 hours, begin rarely after 6 – 24 hours).
d) Other forms of immunologically mediated hypersensitivity (type II, type III according to Coombs and Gell, IgG, IgA, or IgM mediated): Cytopenias, serum sickness, immune complex vasculitis (vasculitis allergica); (reaction time: 24 hours or more).
e) In case of new sensitization under therapy: Typical immunologic sensitization latency 5 – 7 days for types I –IV, with reaction latency of 1 –3 days). Onset of MPE after 7 –10 days. However, the reaction in SJS/TEN, DRESS may occur after weeks, that in autoimmune diseases (e.g., lupus erythematosus) after months, probably due to sensitization only after prolonged exposure (e.g., sensitization or triggering favored by cofactors).

**Table 3.** Typical time interval between start of drug intake and first occurrence of reactions.

**Reaction pattern**	**Time interval**
Urticaria, asthma, anaphylaxis	≤ 1 hour, rarely up to 6 hours after start of exposure
Fixed drug exanthem	≤ 48 hours, rarely later after start of exposure
Maculopapular drug exanthem	4 – 14 days after start of exposure*
AGEP	1 –12 days after start of exposure**
SJS/TEN	4 – 28 days after start of exposure***
DRESS	2 – 8 weeks after start of exposure

AGEP = acute exanthematous generalized pustulosis; SJS = Stevens-Johnson Syndrome; TEN = toxic epidermal necrolysis; DRESS = drug reaction with eosinophilia and systemic symptoms. *In repeat reactions, time interval typically shortened compared to initial reaction. In maculopapular drug exanthem typically reaction after 1 – 4 days, in AGEP, SJS, TEN, DRESS typical time interval after repeat reactions not studied. **For antibiotics mostly 1 – 2 days, for other drugs often 7 – 12 days. ***Sometimes longer for allopurinol.

**Table 4.** Important clinical and anamnestic information for the test plan.

A) Clinical manifestation
– Documentation of clinical manifestations and/or organ systems involved: e.g., skin, airways, cardiovascular system, gastrointestinal tract, liver, kidney.
– Exact description of the clinical-morphological findings (especially in case of skin manifestations/mucosal reactions) additionally photo documentation
– General symptoms: Fever, reduced general condition
– Course of the reaction (onset of the reaction in temporal relation to the drug administration, duration of the reaction, morphological change of the reaction).
– Laboratory findings (e.g., blood count changes such as eosinophilia, thrombocytopenia; liver and kidney values; serum tryptase).
– If necessary, histological findings (especially in the case of blistering skin reactions)
B) Other circumstances of the reaction
– Acute illness at the time of the reaction (e.g., concurrent infectious disease).
– Co-factors that may lower the threshold for an allergic or non-allergic reaction: stress, physical exertion, food intake, alcohol intake, UV exposure, menstruation.
C) Documentation of drugs used in temporal relation to the reaction.
– Indication for drug use
– Trade name
– Mode of application
– Ingredients (active ingredients)
– Duration of application
– Dosage
– Tolerance after previous or during renewed application
D) General history and findings
– Known hypersensitivity reactions (allergy passport)
– Similar reactions without drug application (e.g., natural latex allergy)
– Atopic diseases, food allergy
– Disposing diseases (e.g., asthma, nasal polyps, chronic urticaria, mastocytosis, infections, e.g., HIV, EBV)
– Other relevant past or current medical conditions (including somatoform or mental health conditions)
– Noxious agents: Nicotine, alcohol, drugs
– Current medication (long-term medication)
E) Chronology of the hypersensitivity reaction
– Timing in relation to drug application
– First occurrence
– Course and resolution
– Therapeutic measures and response
F) Diagnosis and pathophysiological classification of the clinical reaction taking into account (see Table 1):
– Morphology and symptoms
– Time course

Note: In the case of multiple reactions, the information for each individual reaction is required.

**Table 5.** Non-irritant skin test concentrations of commonly tested drugs [17].

**Drug or class**	**Skin prick test**	**Intradermal test** ^8^	**Patch test**
**Beta-lactam antibiotics**			
Benzylpenicilloyl-octa-L-lysine	8.6 × 10^-5^ mol/L	8.6 × 10^-5^ mol/L	NA
Sodium benzylpenilloate	1.5 × 10^-3^ mol/L	1.5 × 10^-3^ mol/L	NA
Benzylpenicillin	10,000 UI/mL	10,000 UI/mL	5%
Amoxicillin	20 mg/mL	20 mg/mL	5%
Ampicillin	20 mg/mL	20 mg/mL	5%
Cephalosporins	20 mg/mL^10^	20 mg/mL^10^	5%
**Anticoagulants**			
Heparins^1^	Undiluted^8^	1/10 diluted	Undiluted^8^
Heparinoids^2^	Undiluted^8^	1/10 diluted	Undiluted^8^
**Platinum salts**			
Carboplatin	10 mg/mL	1 mg/mL	NA
Oxaliplatin	1 mg/mL	0.1 mg/mL	NA
Cisplatin	1 mg/mL	0.1 mg/mL	NA
**NSAID**			
Pyrazolone^3^	Suspension^9^	0.1 – 1 mg/mL	10%
Coxibe^4^	Suspension^9^		10%
Other NSAIDs^5^	Suspension^9^	0.1 – 1 mg/mL	10%
**Biologics**			
Adalimumab	50 mg/mL	50 mg/mL	Undiluted^8^
Etanercept	25 mg/mL	5 mg/mL	NA
Infliximab	10 mg/mL	10 mg/mL	NA
Omalizumab	1.25 µg/mL	1.25 µg/mL	NA
**Other**			
Local anesthetics	Undiluted^8^	1/10 diluted	Undiluted^8^
X-ray contrast agent	Undiluted^8^	1/10 diluted	Undiluted^8^
Gadolinium chelates	Undiluted^8^	1/10 diluted	NA
Patent blue	Undiluted	1/10 diluted	NA
Methylene blue	Undiluted	1/100 diluted	NA
Fluorescein	Undiluted^8^	1/10 diluted	Undiluted^8^
Proton pump inhibitors^6^	Undiluted^9^	40 mg/mL	10%
Anticonvulsants^7^	NA	NA	10%
Chlorhexidine digluconate	5 mg/mL	0.002 mg/mL	1%

^1^Heparins: unfractionated heparin, nadroparin, dalteparin, enoxaparin; testing contraindicated in heparin-induced thrombocytopenia. ^2^Heparinoids: danaparoid, fondaparinux. ^3^Pyrazolones: metamizole, propyphenazone, aminopyrine, phenazone, phenylbutazone. ^4^Coxibs: celecoxib, etoricoxib, valdecoxib. ^5^Other nonsteroidal anti-inflammatory drugs: e.g., aspirin, ibuprofen, naproxen, indomethacin, diclofenac, fenoprofen, meloxicam, mefenamic acid, nimesulide. ^6^For lasoprazole and rabeprazole no intravenous solution for intradermal test (IDT), only skin prick test. ^7^For severe reactions, first test with 1%. ^8^Use of commercially available solution for intravenous infusion or subcutaneous injection. ^9^Crushing of the tablet and preparation of a suspension with physiological saline solution. ^10^Only for cefepime 2 mg/mL each. NA = not applicable or no concentration recommended.

**Table 6.** Selection of commercial tests for the determination of specific IgE antibodies against drugs in serum*.

– Ampicilloyl^1^
– Amoxicylloyl^1^
– Cefaclor^2^
– Chlorhexidine^2^
– Gelatin (bovine)^1^ Galactoseα−1,3-galactose (α-gal)^1,3^
– Insulin (Human) ^1^
– Morphine^2^
– Penicilloyl G^1^
– Penicilloyl V^1^
– Pholcodin^2^
– Suxamethonium (succinylcholine) ^2^

*When determining sIgE for drugs, attention must be paid to the validation of the test methods. CE certification requires at least five, FDA registration at least 30 positive patient sera, as well as studies on stability and reproducibility. If these criteria are not met, test reagents may be offered for research purposes. Here, particular attention should be paid to the quality of the deposited literature. In routine diagnostics, no determinations of sIgE should be performed against substances for which no IgE-mediated allergic reactions have been described so far. ^1^CE-certified and FDA-registered; ^2^CE-certified,^3^ α-gal, this is an IgE-reactive sugar epitope which is held responsible for anaphylactic reactions to cetuximab and infusion solutions containing gelatine.
